# EEG-Based Neurocognitive Metrics May Predict Simulated and On-Road Driving Performance in Older Drivers

**DOI:** 10.3389/fnhum.2018.00532

**Published:** 2019-01-15

**Authors:** Greg Rupp, Chris Berka, Amir H. Meghdadi, Marija Stevanović Karić, Marc Casillas, Stephanie Smith, Theodore Rosenthal, Kevin McShea, Emily Sones, Thomas D. Marcotte

**Affiliations:** ^1^Advanced Brain Monitoring Inc., Carlsbad, CA, United States; ^2^Systems Technology, Inc., Hawthorne, CA, United States; ^3^Department of Psychiatry, University of California, San Diego, San Diego, CA, United States

**Keywords:** EEG, event related potentials, sustained attention, driving, HIV, neurodegeneration, driving impairment test, on-road evaluation

## Abstract

The number of older drivers is steadily increasing, and advancing age is associated with a high rate of automobile crashes and fatalities. This can be attributed to a combination of factors including decline in sensory, motor, and cognitive functions due to natural aging or neurodegenerative diseases such as HIV-Associated Neurocognitive Disorder (HAND). Current clinical assessment methods only modestly predict impaired driving. Thus, there is a need for inexpensive and scalable tools to predict on-road driving performance. In this study EEG was acquired from 39 HIV+ patients and 63 healthy participants (HP) during: 3-Choice-Vigilance Task (3CVT), a 30-min driving simulator session, and a 12-mile on-road driving evaluation. Based on driving performance, a designation of Good/Poor (simulator) and Safe/Unsafe (on-road drive) was assigned to each participant. Event-related potentials (ERPs) obtained during 3CVT showed increased amplitude of the P200 component was associated with bad driving performance both during the on-road and simulated drive. This P200 effect was consistent across the HP and HIV+ groups, particularly over the left frontal-central region. Decreased amplitude of the late positive potential (LPP) during 3CVT, particularly over the left frontal regions, was associated with bad driving performance in the simulator. These EEG ERP metrics were shown to be associated with driving performance across participants independent of HIV status. During the on-road evaluation, Unsafe drivers exhibited higher EEG alpha power compared to Safe drivers. The results of this study are 2-fold. First, they demonstrate that high-quality EEG can be inexpensively and easily acquired during simulated and on-road driving assessments. Secondly, EEG metrics acquired during a sustained attention task (3CVT) are associated with driving performance, and these metrics could potentially be used to assess whether an individual has the cognitive skills necessary for safe driving.

## Introduction

Driving is an essential aspect of maintaining health, independence and quality of life as individuals age (Ball et al., 1998). Those who voluntarily avoid driving due to perceived age-related sensory or cognitive deficits often suffer substantial consequences such as decreased mobility, increased dependency, social isolation, depression, and higher incidence of nursing home placement (Marottoli et al., 1997, 2000; Fonda et al., 2001; Ragland et al., 2005; Freeman et al., 2006; Czigler et al., 2008; Choi et al., 2013). Driving requires a myriad of cognitive functions including attention, visuospatial processing, psychomotor integration, adequate processing speed, and executive function (Kellison, 2009). Normal aging, in the absence of any neurological or psychiatric disease, can lead to declines in these cognitive abilities increasing the risk for an automobile collision (Brayne et al., 2000; Ball, 2009). However, the aging process and its effects on driving performance vary significantly between individuals (Ball, 2009). It has been suggested that specific age-related functional impairments, and not age itself, put one at risk for impaired driving (Ross et al., 2009). Overall, older drivers as a group incur the highest number of fatalities per mile driven compared to other age groups (although the physical frailty of older individuals contributes significantly to this mortality rate) (Tefft, 2017). In addition to normal aging, functional deficits associated with neurodegenerative diseases (NDDs) such as Alzheimer's (AD), Mild Cognitive Impairment (MCI), or HIV-associated neurocognitive disorders (HAND) may affect driving performance. While NDD patients are more likely to be at-risk drivers, research suggests that memory deficits alone may not necessarily lead to unsafe driving (Carr et al., 1998; Marcotte et al., 1999, 2004; Silverstein et al., 2002; Charlton et al., 2003; Duchek et al., 2003; Uc et al., 2004, 2005; Man-Son-Hing et al., 2007; Frittelli et al., 2009; Wadley et al., 2009; Kawano et al., 2012). Cognitive impairments that affect driving, such as visuospatial processing deficits often found in patients with MCI or HAND, may be subclinical and unobserved by the patient themselves or their friends and family (Cysique et al., 2009; Chiao et al., 2013). Therefore, driving impairment cannot be established using only age and/or a NDD diagnosis.

In the United States, legal requirements for elderly drivers vary greatly from state to state. Some states have no safety-related policies for older drivers, whereas other states may have limited requirements for elderly individuals. For example, license renewal in California for those over the age of 70 may require a vision and/or written test, and in rare cases an on-road evaluation is administered (Department of Motor Vehicles, 2018a). Other states like Connecticut and Delaware have no age-related safety policies in place. Driver's licenses in these states need to be renewed every 6–8 years for all drivers regardless of age, often with no functional assessment required (Department of Motor Vehicles, 2018b,c).

Physicians have a responsibility to identify patients of all ages that might be considered at-risk drivers. However, they are often reluctant to take action due to privacy concerns and/or the severe impact their intervention could have on the patient's quality of life that results from the loss of a driver's license. Currently, there is no definitive diagnostic test for physicians to administer that identifies at-risk drivers, but individuals deemed potentially high risk may be referred for neuropsychological testing. The relationships between on-road driving performance and standard neuropsychological tests are modest, particularly in patients with mild to moderate cognitive decline or those recovering from trauma, surgery or treatments such as chemotherapy (Withaar et al., 2000; Reger et al., 2004; Leproust et al., 2008; Classen et al., 2009). The most reliable method of evaluating driving impairment is an on-road test with a DMV-certified driving examiner, but annual on-road driving evaluations for all seniors, or even just those with clinically diagnosed cognitive impairments, are neither practical nor economical (Schanke and Sundet, 2000; Kay et al., 2008; Versijpt et al., 2017). Therefore, there is a need for inexpensive and sensitive tests to predict on-road driving impairment.

This study investigated the use of simultaneous electroencephalogram (EEG) and electrocardiogram (ECG) measurement in a population of healthy participants and HIV+ patients (>55 years old) during a test of sustained attention and processing speed. The combination of EEG, ECG, and behavioral performance metrics derived from the 3CVT were previously proven highly sensitive and specific in quantifying daytime drowsiness associated either with sleep deprivation in healthy participants or in sleep disordered patients, predicting susceptibility to sleep deprivation, and assessing neurocognitive deficits in patients with Parkinson's disease (PD), AD, MCI, and sleep disorders (Westbrook et al., 2002; Berka et al., 2006, 2007, 2009; Pojman et al., 2009a,b; Johnson et al., 2010, 2011; Waninger et al., 2018). The 3CVT evaluates sustained attention, visuospatial processing speed, and decision-making. These cognitive abilities are relevant to driving performance and prior work suggests that EEG metrics obtained during 3CVT were sensitive to improvements in cognition as a result of successful interventions for both sleep deprivation and sleep disorders (Westbrook et al., 2002; Berka et al., 2006, 2007, 2009; Pojman et al., 2009a,b; Johnson et al., 2010, 2011; Stoiljkovic et al., 2018). In addition to 3CVT, EEG and ECG were also acquired during both a simulated driving scenario and an on-road driving evaluation to conduct an exploratory analysis to assess any potential real-time neurophysiological changes associated with driving performance. Specifically, differences in the early (P200) and late (LPP) components evoked by the 3CVT have been associated with differences in cognitive abilities such as selective attention, memory, and decision-making. Since the 3CVT EEG metrics were previously shown to be associated with neurocognitive deficits in cognitively impaired populations, the investigators hypothesized that these metrics could be useful in distinguishing Safe and Unsafe drivers.

Physiological (heart rate, heart rate variability, skin conductance, and respiration) and neurophysiological (EEG) measures have long been used to unobtrusively assess the psychophysiological correlates of driving performance during simulated and on-road driving. Characteristic changes in EEG Power Spectral Densities (PSDs) have been associated with real-time changes in driving performance, phasic task demands, multiple domains of workload, and drowsiness (Zwinkels et al., 1990; de Waard and Brookhuis, 1991; Brookhuis and de Waard, 1993; Rookhuis et al., 1993; Mitler et al., 1997; Lei and Roetting, 2011; Dijksterhuis et al., 2013). Similarly, heart rate and heart rate variability have proven useful in measuring dynamic changes in cognitive demand during driving (Brookhuis et al., 1991; Mulder, 2004; Mehler et al., 2009, 2012). Several recent reports suggest the potential utility of real-time EEG-based algorithms to detect driver drowsiness and inattention. Continuous monitoring of EEG and heart rate data during driving provides excellent temporal resolution and offers the potential for identifying driver fatigue early enough to intervene and prevent sleep onset. Several recent reports suggest the potential utility of real-time EEG-based algorithms to detect driver drowsiness and inattention (Ajinoroozi et al., 2016; Perrier et al., 2016; Hajinoroozi et al., 2017). Several challenges remain for the implementation of integrated driver monitoring systems including: obtaining high quality EEG and ECG with unobtrusive sensor systems, validating and implementing the real-time algorithms to achieve accurate identification of fatigue or inattention, and determining the optimal approach to interventions during driving (Dong et al., 2010). Another important consideration is the generalizability of the algorithms across all age groups, as the majority of published results use algorithms that have been designed and tested on college age research participants. These EEG-based algorithms are used to monitor real-time changes during driving. To date, EEG metrics have not been used to predict driving performance in elderly individuals with or without cognitive impairment.

As normal aging is associated with changes in cognitive abilities related to driving, normal aging also affects EEG signals. Older individuals show a decrease in power in the alpha band (8–13 Hz) and decreased amplitude of ERP components, particularly the P300 and Late Positive Potential (LPP) (De Gennaro et al., 2005; Polich and Corey-Bloom, 2005; Olichney et al., 2008; Vecchio et al., 2013; López et al., 2014; Ishii et al., 2017). Older drivers are also more likely to exhibit EEG based signs of fatigue and distraction that increase risks of driving errors (Johansson, 1997). In patients diagnosed with Alzheimer's disease, the most commonly reported findings for resting-state EEG are: a shift of the power spectrum to slower frequencies (i.e., increased delta and theta specifically over the temporal-parietal regions; decreased alpha, beta, and gamma) (Bonanni et al., 2008; Jelic and Kowalski, 2009; Dauwels et al., 2010a,b; Tsolaki et al., 2014). Patients with AD also display prolonged latencies and diminished ERP amplitudes and these cognitive-evoked measures do tend to correlate better with severity of cognitive impairments (Polich and Corey-Bloom, 2005; Garn et al., 2014). The EEG power shifts and ERP differences in AD are primarily associated with memory related functions. Additionally, patients with HIV (with a subset of those potentially having HAND) exhibited decreased amplitude and increased latency of the P300 and the Late Positive Potential (LPP) components compared to healthy controls (Polich et al., 2000; Polich and Basho, 2002; Chao et al., 2004; Bauer, 2011; Olichney et al., 2011; Papaliagkas et al., 2011). To date, these studies have not directly examined the relationship between EEG metrics associated with aging or cognitive impairment and driving competencies.

This paper contributes to the field by: (1) establishing the link between neurophysiological measures obtained during computerized neurocognitive assessments and on-road driving performance, (2) evaluating older adults (>55 years old) and individuals with a condition that can lead to cognitive impairment (HIV+). As such, this research offers the potential to provide a standardized methodology for predicting driving impairment due to disease related causes or natural aging.

## Materials and Methods

### Participants

Sixty-three healthy participants (HP) (age 55–87 years, mean = 65 ± 8.2 years, 49.2% male) and 39 HIV+ patients (age 55–74 years, mean = 61 ± 4.7 years, 87.1% male) were enrolled in the study. The groups did not differ in years of education (HIV+: 9–20 years of education, mean = 15.5 ± 2.9; HP: 10–21 years of education, mean = 15.6 ± 2.7). HIV+ patients were primarily recruited from the University of California, San Diego HIV Neurobehavioral Research Program (UCSD HNRP) and healthy participants from the surrounding San Diego community using flyers and handouts.

Participants were selected after an initial telephone screening to determine their eligibility including the capability to provide informed consent to cognitive testing, simulator testing, and an on-road driving evaluation. Participants were included only if they possessed a current driver's license which was confirmed by the California Department of Motor Vehicles (CA DMV) on the day of their visit.

Additional exclusion criteria were: a history of loss of consciousness >30 min, current substance dependence, psychosis, diagnosis of a cardiovascular, sleep, or pulmonary disorder, and central nervous system opportunistic infections or neurologic disease other than HIV infection, reported diagnoses of Attention Deficit Hyperactivity Disorder (ADHD) or anxiety related disorders. All HIV+ individuals were on anti-retroviral therapy to control viral load, and healthy participants were excluded for all medication except for over the counter drugs and drugs for hypertension, diabetes, arthritis (non-opioid pain medication), and mild to moderate depression. The HIV+ populations used for this study were taking the following medications: 15 on antidepressants, eight on benzodiazepines, two on antipsychotics, three on anxiolytics, three on narcotics, and one on an anticoagulant. Urine toxicology (7-panel) and breathalyzer evaluations were also collected from all participants prior to starting the study visit. If either test was positive or the participant acted in a manner suggesting intoxication, he/she was rescheduled, or withdrawn from the study.

Three participants who signed informed consent forms and began the study protocol were excluded from all analyses due to a positive urine test for methamphetamine, and one additional participant was excluded due to being severely cognitively impaired despite a negative HIV status. Protocols were approved by both the UCSD IRB and Sharp IRB (IRBANA).

### Procedures

All participants completed neuropsychological (NP) testing and Advanced Brain Monitoring's (ABM) 3-Choice Vigilance Task (3CVT) as well as driving simulations (a screening drive, and subsequent challenge drive). A subset of the participants from the HIV+ (N=20) and HP (N=30) groups also completed an on-road driving evaluation (see below). EEG was collected concurrently using ABM's STAT™ X10 EEG sensor headset during all three tasks: 3CVT, simulated driving, and the on-road driving evaluation. The X10 is a battery-powered, lightweight, easy-to-apply wireless EEG system that acquires 9 channels of EEG (Fz, F3, F4, Cz, C3, C4, P3, P4, POz, referenced to linked mastoids), and ECG. It uses passive, Ag/AgCl electrodes printed on PET strip flex circuit cables. A piece of single-use foam filled with conductive cream (Synapse by Kustomer Kinetics) was attached to the strip over each electrode site in order to make contact with the scalp. Impedances were measured and all channels were considered acceptable at or below 40 kOhms. Amplification and the A/D conversion was done adjacent to the electrode sites, allowing for high-quality data to be collected with higher than traditional impedance cut-offs. Data were sampled at 256 Hz with a high band pass at 0.1 Hz and a low band pass, fifth order filter, at 100 Hz obtained digitally with sigma-delta 16-bit A/D converters. Data were transmitted wirelessly via Bluetooth to a host computer, where acquisition software then stored the psychophysiological data.

#### Cognitive and Medical Assessment

Cognitive status was successfully obtained through NP testing for 85 of the 102 participants (29 HIV+ and 56 HP), determined using either the HNRP NP assessment battery (56% of cohort) or the NIH Toolbox Cognition module (44% of cohort) (Berka and Marcotte, 2017). For this subset of participants, 34% of the HIV+ group was classified as impaired and 27% of the healthy participants were classified as impaired based on the NP testing, meaning there were no group differences in cognitive status due to HIV status. Impairment was defined for the toolbox as a T score of <40 on two of the tests, and for the NP assessment as a global deficit score of <0.5. For all participants, HIV status was confirmed through a finger stick blood test.

#### 3CVT and EEG Measures

All participants were administered 3CVT, with concurrent EEG recording to assess neurocognitive functions. The 3CVT incorporates features of the most common measures of sustained attention, such as the Continuous Performance Test, Wilkinson Reaction Time, and the PVT-192 (Riccio et al., 2001; Sateia, 2003). The 3CVT requires subjects to discriminate one primary Target (triangle shape ▴, 70% of trials) from Non-Target (triangle shape upside down ▾, 15% of trials). The remaining 15% of the trials were used as Distracters (presenting a diamond shape: ♦) to increase the task complexity but are not included in the final Event Related Potential analysis. The test is 20-min long, during which 376 images are presented for a duration of 0.2 s each. A training period is provided prior to the start to minimize practice effects (Levendowski et al., 2000, 2001). The 3CVT challenges the participant's ability to sustain attention by increasing the inter-stimulus interval (ISI) across four, 5-min quartiles. During the first quartile, the ISI ranges between 1.5 and 3 s, increasing up to 6 s during the second quartile, and up to 10 s during the third and fourth quartiles.

##### ERP Measures

For the 3CVT task, raw EEG signals were filtered between 0.1 and 50 Hz using a Hamming windowed Sinc FIR filter (0.1 Hz transition band). For each event type, EEG data were epoched from 1 s before and 2 s after the stimulus onset. The baseline was adjusted using data from 100 ms before the stimulus onset. Artifacted epochs were detected and excluded using automated algorithms (EEGLAB software) (Delorme and Makeig, 2004). Outliers were detected based on kurtosis of signal distribution (kurtosis >5 standard deviation), joint probability of values in an epoch given the whole data set (thresholded at 5 standard deviation), and unusual spectral patterns of epochs (with power spectrum 35 dB higher or lower than the baseline in the frequency range of 20–30 Hz). To exclude trials contaminated by ocular artifacts, trials were rejected if the absolute value of the EEG amplitude in any channel exceeded 100 microvolts during a window of 50 ms pre-stimulus onset to 750 ms post-stimulus onset. A minimum of 15 clean trials for each of the stimulus subtypes in 3CVT (Target and Non-Target) were required to be included in the analysis of that subtype. Grand average ERPs in each condition and trial type were calculated using a weighted average with the number of ERPs in each condition as the weights. For each participant, ERPs were measured using the average of the signal during a window of 180–220 ms post-stimulus onset for the P200 component, and the late positive potential (LPP) was measured using the average of the signal during a window 300–700 ms post-stimulus onset.

#### Simulated Driving

**Figure 1:**
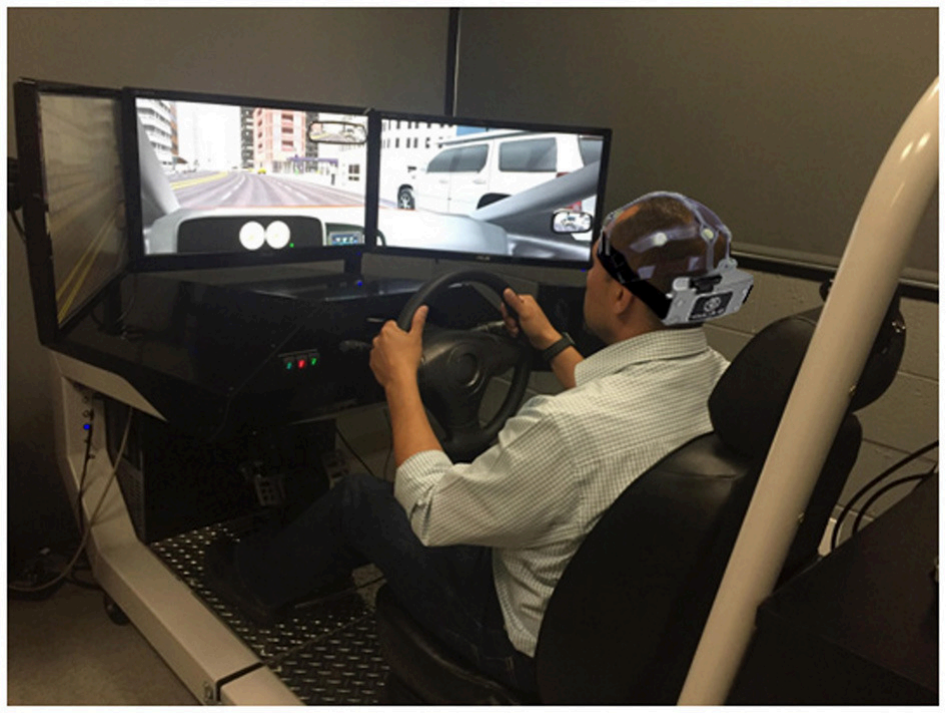
STISIM M300WS console with participant (written informed consent obtained).

Participants completed two simulated driving scenarios: an initial screening and a challenge. Seventy-eight percent of eligible participants were able to complete both scenarios. The remaining 22% were unable to complete both scenarios, primarily due to mild to severe motion sickness. To mitigate motion sickness, the driving scenario was split into three sessions with breaks in between. A STISIM M300WS Console driving simulator (System Technology Inc., Hawthorne, CA, USA) was used for both sets of driving simulations (Figure 1). The screening drive is a practice session of approximately 15 min given in order to familiarize participants with the driving simulator. Following the Screening Drive, participants began the Challenge Drive, which is a longer (3, 10-min segments, 30 min total), more complex drive assessing a range of abilities. Participants were instructed to complete the Challenge Drive while following traffic laws. The Challenge Drive was designed to be a surrogate for measuring on-road driving performance.

The Challenge Drive consisted of monotonous, uneventful, and low-load driving scenarios as well as highly demanding events such as busy intersections, crash avoidance, and unprotected turns. Busy sections were interspersed throughout the simulation run and lasted for 4–5 min. For example, one complex segment required the driver to avoid and pass slow moving cars while driving through dense fog. Once the fog lifted, the driver entered a city scene where a van was parked in the left lane and two pedestrians suddenly stepped into the road from in between two parked cars. Other highly engaging events included passing through a narrow construction zone with many barriers, avoiding cars suddenly entering the roadway, making left turns in front of oncoming traffic, passing slow moving trucks on a two-lane highway with oncoming traffic, and avoiding pedestrians stepping into the roadway without warning. The non-challenging sections consisted of stretches of highway where no other cars were present and no challenging events were triggered.

**Figure 2:**
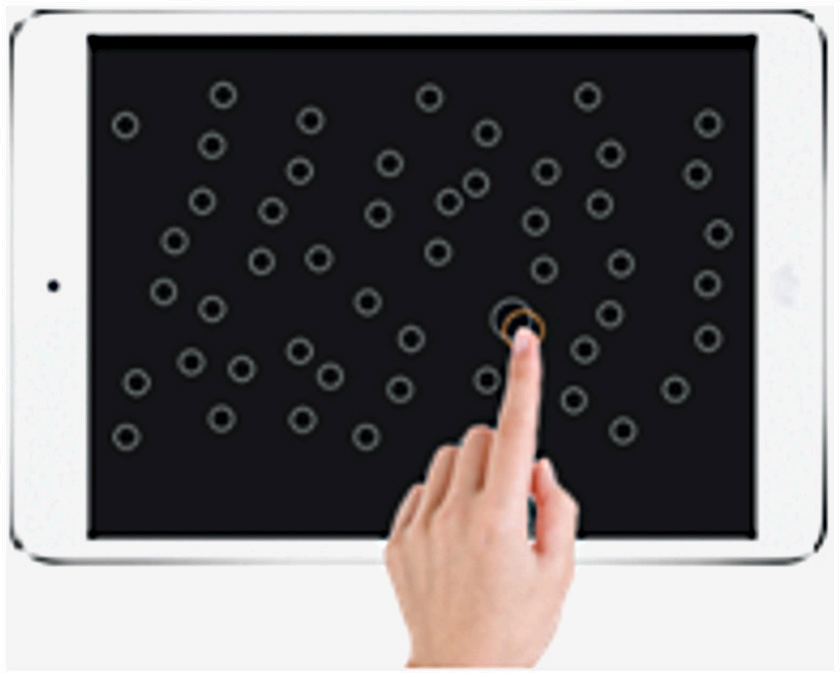
Example of a participant identifying correct, target circle.

The Challenge Drive also contained a divided attention task called the Surrogate Reference Task (SuRT), aimed at examining distracted driving. The SuRT was initiated by an auditory cue (phone ringing) and required the participant to look down and to their right, forcing them to take their eyes entirely off the roadway to perform this secondary task, much like using a GPS or infotainment system. Participants were required to identify a circle that was different in size from other circles on the screen of a tablet (Figure 2). The easy, medium, and hard trials of this task were differentiated by the difference in size between the target and distractor circles. The target circle radii remained 20.7 mm for all three trials, while the distractor circle radii increased from 10.4 to 13.8 to 17.4 mm. Throughout this task, the simulation consisted of a two lane freeway without turns, a speed limit of 65 MPH, and no cars in either direction. Outcomes of interest included swerving [standard deviation of lateral position (SDLP)], speed maintenance (including variability) as well as accuracy and reaction time on the secondary task.

#### On-Road Driving Evaluation

A subset of 50 participants (age 55–79 years, mean = 62 ± 6.6, 66% male, 40% HIV+) who completed the neurocognitive testbed and the driving simulator were selected to complete the on-road drive. Only 50 were selected due to time and budget restraints; selected participants must have completed the 3CVT and driving simulator scenarios. The on-road driving route was approximately 12 miles and required, on average, 45 min to complete (Supplementary Figure 1). It was conducted by the Sharp Rehabilitation Services Driving Program using a standardized approach with excellent inter-rater reliability (Cohen's *K* = 0.86) and established sensitivity to HIV-related driving changes. A DMV-certified driving examiner was positioned in the front passenger seat of a dual-brake automobile; an occupational therapist (OT) and ABM technician (taking detailed notes about the driving safety and performance as well as monitoring the EEG signals) observed the drive in the rear seats. Participants were instructed to drive through residential and commercial areas, across controlled and uncontrolled intersections, and on freeways (including multiple merges). The participants followed single and multi-step directions (e.g., “Make the next available right turn… In three traffic lights, make a left turn”) throughout the duration of the drive.

### Evaluating Driving Performance

In order to evaluate driving performance participants were divided into groups of “Good” or “Poor” drivers based on performance in the simulator and “Safe” or “Unsafe” drivers based on on-road performance. The following sections describe this group assignment process.

#### On-Road Performance

Both the driving examiner and OT evaluated the drive in two ways. First, 186 scoring criteria for correctly performing traffic checks, maintaining lane position and speed, yielding when appropriate, etc. were assigned either a zero for pass, or a one for fail. Second, participants were given an overall score of 1 (excellent) through 5 (recommends they should not be driving) (Supplementary Table 1).

Each evaluator independently completed the pass/fail scores during the drive, and assigned an overall score after the conclusion of the drive. The driving instructor and OT would then arrive at a consensus evaluation for the overall score as well as a consensus regarding individual pass/fails. In addition, the OT documented critical errors in the form of physical or verbal interventions. Physical interventions included using the passenger-side brake and grabbing the wheel, while verbal interventions included any additional instructions or warnings that were not part of the scripted directions. Each driver was designated Safe or Unsafe based on the consolidated raters scores, comments, critiques, observations, and critical errors. Thirty-five of the 50 drivers were designated Safe (70%) and 15 drivers were designated Unsafe (30%).

#### Driving Simulator Performance

Individual mistakes over the course of the challenge drive were counted and given weights to generate a weighted score as follows:

- 3 pts for a collision with another vehicle
- 2 pts for running stop signs or red lights
- 1 pt for speed exceedances and lane marker collisions (e.g., a traffic cone in a construction zone)
- 0.5 pts for crossing over the center dividing line or crossing into the right shoulder without causing a collision.

**Figure 3:**
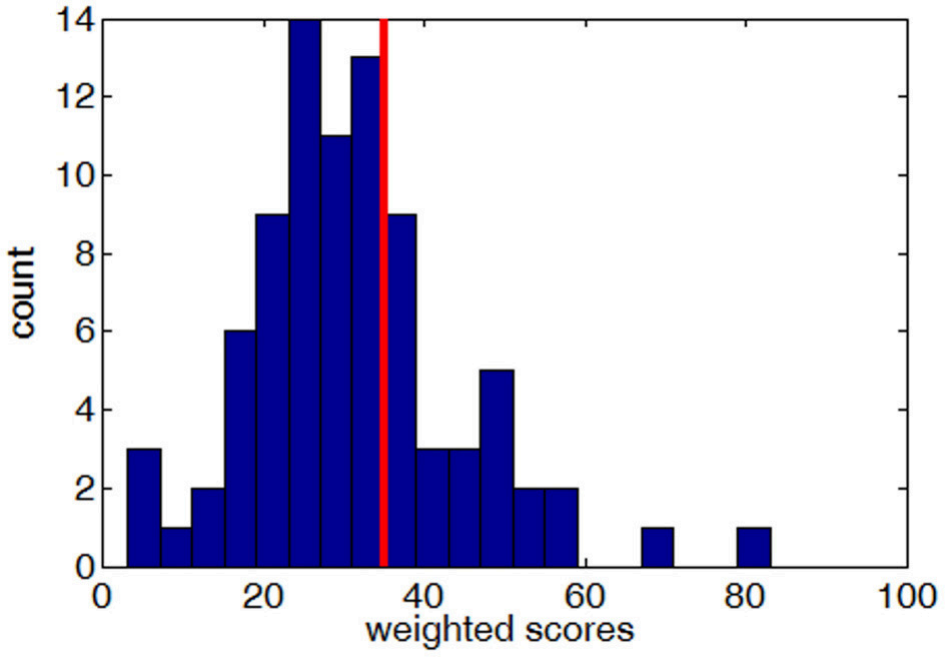
The distribution of weighted scores (higher scores indicate worse performance) across all subjects who completed the simulated drive, with the red line showing the cut-off threshold of 35.

Using these weights, a total weighted score was computed for each participant who completed the simulated drive. This weighted score was used to divide drivers into either Good or Poor groups. Drivers with a weighted score of 35 or more designated as Poor. This threshold of 35 was chosen to result in 70/30% Good/Poor ratio to match the Safe/Unsafe ratio observed during the on-road drive (see On-Road Performance). Figure 3 shows the distribution of weighted scores for all participants who completed the simulated driving scenario.

### Predicting Driving Performance

A linear discriminant function (LDF) was designed to classify Safe vs. Unsafe drivers using EEG ERP measures (P200 and LPP for both Target and Non-Target trials across all channels) obtained during the 3CVT test. The variables used for the LDF were selected through a step-wise algorithm in a logistic regression analysis. The classifier was evaluated using a leave-one-out cross validation method.

## Results

EEG and behavioral measures were computed for all three tasks (3CVT, simulated driving, and on-road evaluation). Performance in the driving simulator was used to group subjects into either Good or Poor (section Driving Simulator Performance), and on-road driving performance was used to designate subjects as either Safe or Unsafe (section On-Road Performance). To investigate the relationship between each behavioral/EEG measure and driving performance, these measures were averaged across the Safe (or Good) groups and were compared to the average of the Unsafe (or Poor) groups.

To investigate the relationships between HIV seropositivity and driving performance, chi-square tests of independence were performed for simulated and on-road driving groups. The proportion of Good vs. Poor (60.6 vs. 39.4%) drivers in the HIV+ group was not significantly different than that of the HP group (69.2 vs. 30.8%) [**χ2** (1, *n* = 85) = 0.34, *p* = 0.56]. Similarly, the proportion of Safe vs. Unsafe (60.0 vs. 40.0%) drivers in the HIV+ group was not significantly different than that of the HP group (76.6 vs. 23.4%) [**χ2** (1, *n* = 50) = 0.89, *p* = 0.34]. Therefore, driving performance both in the simulator and on-road was determined to be independent of HIV status in this population.

### Behavioral Measures

Behavioral measures included simulated driving performance, on-road driving performance, and Reaction Time (RT)/Accuracy for the 3CVT, as described in sections Driving Simulator Performance, On-Road Performance, and 3CVT and EEG Measures, respectively.

#### 3CVT Behavioral Measures as Predictors of Driving Performance

**Figure 4:**
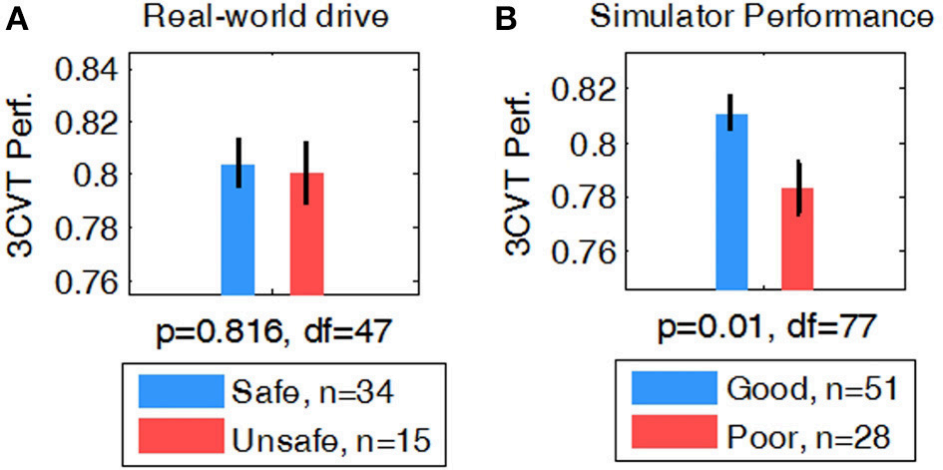
Comparison of F-measure for **(A)** Safe vs. Unsafe and **(B)** Good vs. Poor.

Behavioral measures during the 3CVT attention task were computed for each participant including RT, Accuracy (percent correct), and a combined measure of performance (F-measure, i.e., a harmonic mean of normalized accuracy and reaction time) (Stikic et al., 2011). A student's t-test was used to determine whether group averages of 3CVT behavioral measures were different for Safe/Unsafe (on-road drive) and Good/Poor (driving simulator) drivers. F-measure showed no significant difference in performance between Safe and Unsafe drivers (*p* = 0.81, df = 47) (Figure 4A). However, Good drivers in the simulator had significantly higher performance compared to Poor drivers (*p* < 0.01, df = 77) (Figure 4B).

#### Driving Simulator

Throughout the driving simulation, there was high variability between subjects in speed, speed deviation, SDLP, and time to collision as individuals navigated the various complex segments with varying approaches. For example, Supplementary Table 2 shows the high variance of speed between subjects for each block. Although participants were instructed to follow the rules of the road, the completion time for each segment of the driving scenario varied widely between participants. Because of the high between- and within- subject variability of these metrics, driving performance in the simulator was quantitatively computed using the variables described in section Driving Simulator Performance. To assess the relationship between on-road driving performance and simulator performance, a chi-squared test of independence was performed. 72.7% of Safe drivers were Good in the simulator and 71.4% of Unsafe drivers were Poor in the simulator [**χ2** (1, *n* = 47) = 6.23, *p* = 0.01].

##### SuRT Performance in Driving Simulator to Predict Simulator/On-Road Driving Performance

**Figure 5:**
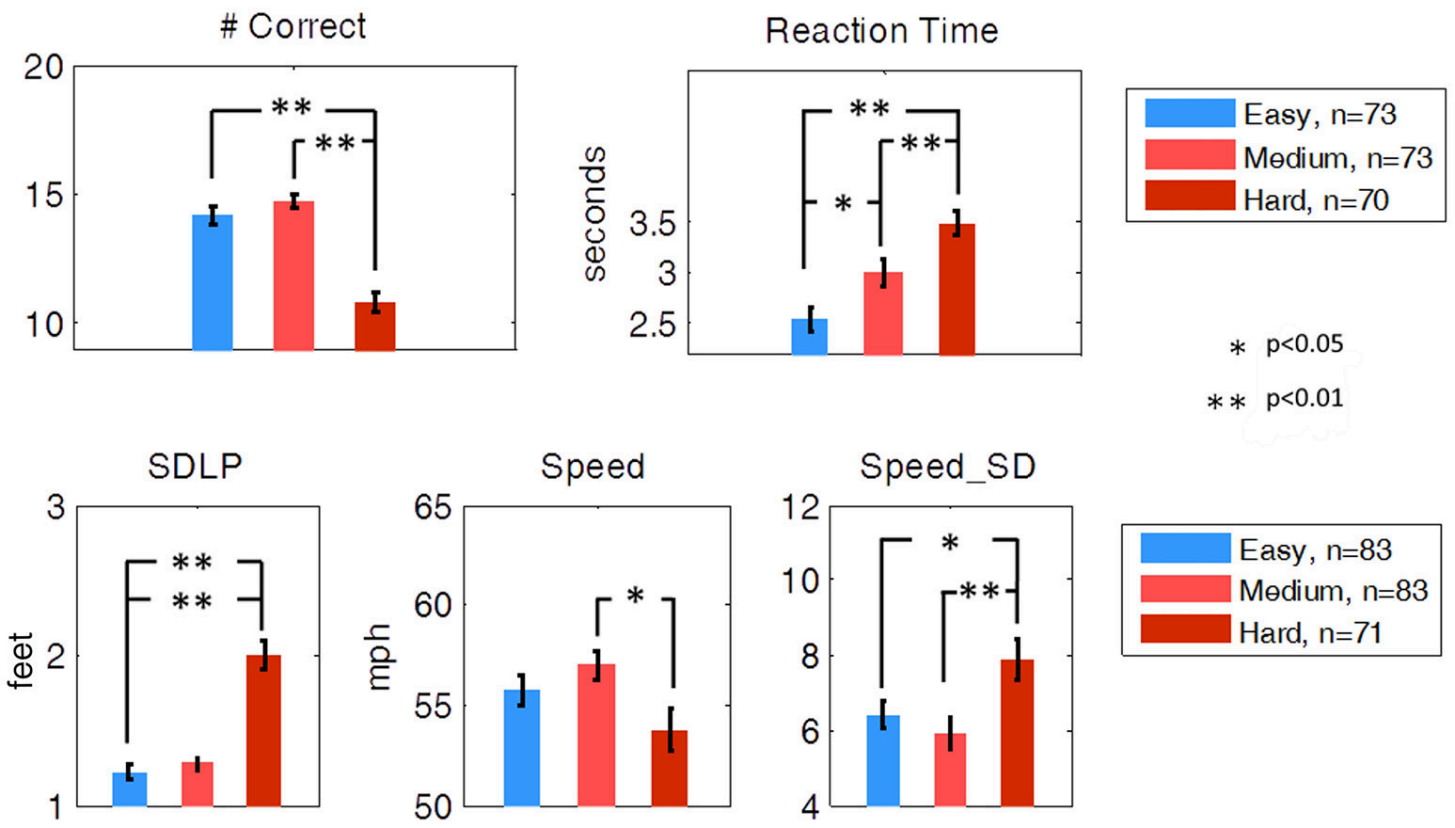
Secondary task performance and driving performance during the SuRT Easy, Medium, and Hard task. Participants performed worse, as indicated by all metrics except average speed, on the most difficult SuRT task.

The mean Number Correct and mean Reaction Time for each of the three difficulty levels of the secondary task are illustrated in Figure 5. Students' *t*-tests revealed that no significant difference in Number Correct from easy to medium was present, but Number Correct did differ significantly between medium and hard (*t*-test, df = 141, *p* < 0.01), and easy to hard (*t*-test, df= 141, *p* < 0.01). Mean Reaction Time significantly increased from easy to medium (*t*-test, df = 143, *p* < 0.05) and medium to hard (*t*-test, df = 141, *p* < 0.01).

Ideal driving behavior during the SuRT would be characterized by a low rate of swerving (low SDLP), an average speed close to the speed limit (65 MPH), and a low rate of speed deviation. SDLP significantly increased from easy to hard (*t-*test, df = 141, *p* < 0.01) and from medium to hard (*t-*test, df = 141, *p* < 0.01). Speed deviation significantly increased from easy to hard (*t-*test, df = 141, *p* < 0.05) and medium to hard (*t-*test, df = 141, *p* < 0.01). Average Speed decreased from medium to hard (df = 141, *p* < 0.05).

While the SuRT task proved to be useful in measuring the effect of multitasking on driving behavior, neither SuRT driving performance nor secondary task performance were significantly different for Good vs. Poor (simulator) or for Safe vs. Unsafe (on-road) drivers.

#### On-Road Drive

The overall Safe and Unsafe driver's scores were computed as described in section On-Road Performance and were used for group comparisons.

### Association Between 3CVT EEG ERP Measures and Driving Performance

**Figure 6:**
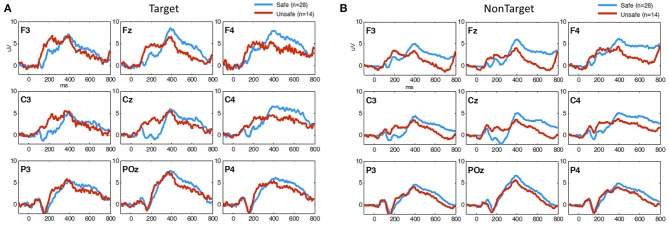
Grand Average ERP plots (averaged across participants) for **(A)** Non-Target and **(B)** Target trials during 3CVT task plotted for Safe (blue)/Unsafe (red).

EEG measures obtained during 3CVT were compared for each group in order to discover any potential associations between 3CVT EEG measures and driving performance measures. Figure 6 shows the grand average ERPs for 3CVT Non-Target trials (left) and Target trials (right) plotted to compare the Safe and Unsafe drivers. On average, Unsafe drivers exhibit higher amplitudes at 200 ms post-stimulus onset and lower amplitude from 300 to 700 ms post-stimulus onset.

**Figure 7:**
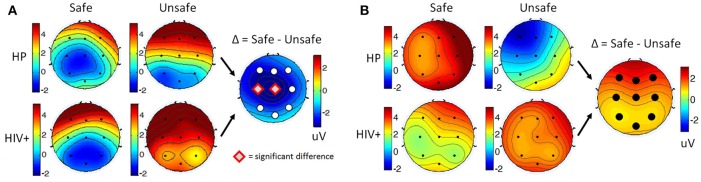
Topographical maps of **(A)** the average P200 component in Non-Target ERP trials (left panel) and **(B)** average LPP component in Target trials (right panel) plotted for all subgroups: Safe/HP, Safe/HIV+, Unsafe/HP and Unsafe/HIV+. In each panel, the difference plot between total Safe and Unsafe groups is shown on the right side. Channels with significant differences between the two groups (*t*-test, *p* < 0.05) are marked with a diamond sign.

For each participant, ERPs were measured using the average of the signal during a window of 180–220 ms post-stimulus onset for the P200 component, and the late positive potential (LPP) was measured using the average of the signal during a window 300–700 ms post-stimulus onset. Safe drivers exhibited a significantly smaller P200 over the left central region for Non-Target trials compared to Unsafe drivers (Figure 7A). HP Safe drivers exhibited a significantly larger LPP over the left frontal region compared to HP Unsafe drivers for Target trials (Figure 7B). There was no significant difference between HIV Safe and HIV Unsafe in terms of LPP amplitude (Figure 7B). Additionally, there was no significant difference in LPP amplitude when comparing Safe and Unsafe drivers from both groups. Table 1 summarizes the significant findings. The difference in the P200 and LPP components between Safe and Unsafe drivers are listed for both trial types (Target and Non-Target) and for all channels in Supplementary Table 3.

**Table 1:** Average P200 components for all groups and subgroups based on on-road driving performance.

			**P200 avg (NonTarget)**	
			**Mean** **±** **SEM (uV)**	
**Condition**	**Group**	***n***	**Cz**	**C3**
HP	Safe	19	−1.07 ± 0.96	−0.31 ± 0.80
	Unsafe	7	2.76 ± 1.36	2.30 ± 0.95
HIV+	Safe	9	1.32 ± 1.89	2.37 ± 1.35
	Unsafe	7	3.34 ± 1.94	3.97 ± 1.98
All	Safe	28	−0.30 ± 0.90	0.55 ± 0.72
	Unsafe	14	3.05 ± 1.14	3.14 ± 1.08
HP	Δ = Safe-Unsafe	26	−3.83^*^	−2.62
HIV+	Δ = Safe-Unsafe	16	−2.02	−1.60
All	Δ = Safe-Unsafe	42	−3.35^*^	−2.59^*^

*Significant differences (t-test, p < 0.05) are marked with asterisk*.

**Figure 8:**
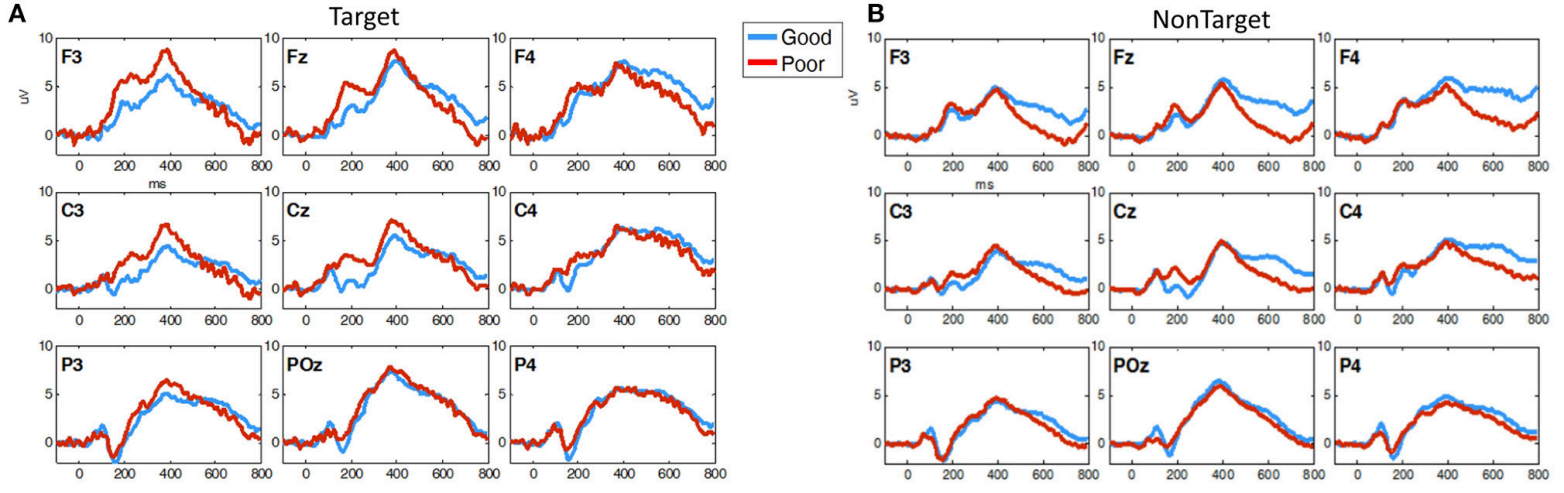
Grand average ERP plots (averaged across participants) for **(A)** Non-Target and **(B)** Target trials during 3CVT task plotted for Good (blue)/Poor (red).

Figure 8 shows the grand average ERPs for 3CVT Non-Target trials (left) and Target trials (right) plotted to compare the Good and Poor drivers in the simulator. On average, Poor drivers exhibit higher amplitudes at 200 ms post-stimulus onset, and lower LPP amplitude from 300 to 700 ms post-stimulus onset.

**Figure 9:**
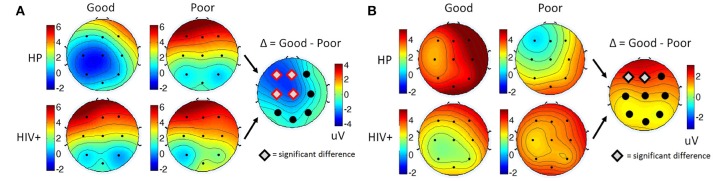
Topographical maps of **(A)** the average P200 component in Non-Target ERP trials (left panel) and **(B)** average LPP component in Target trials (right panel) plotted for all subgroups: Good/HP, Good/HIV+, Poor/HP and Poor/HIV+. In each panel the difference plot between total Good and Poor groups is shown on the right side. Channels with significant differences between the two groups (*t*-test, *p* < 0.05) are marked with a diamond shape.

Overall, Poor drivers had a significantly higher P200 over left frontal-central channels (Figure 9A) and a significantly lower LPP amplitude over left frontal channels (Figure 9B) compared to Good drivers. Table 2 summarizes the P200 findings and Table 3 summarizes the LPP findings for all significant channels. The difference in the P200 and LPP components between Good and Poor drivers are listed for both trial types (Target and Non-Target) and for all channels in Supplementary Table 4.

**Table 2:** Average P200 amplitude for all groups and subgroups based on simulator driving performance.

		***n***	**P200 avg (NonTarget)**			
			**Mean** **±** **SEM (uV)**			
			**Cz**	**C3**	**Fz**	**F3**
HP	Good	30	−0.84 ± 0.73	−0.63 ± 0.58	1.15 ± 0.79	1.19 ± 0.68
	Poor	13	2.92 ± 0.90	2.88 ± 0.78	4.70 ± 1.01	5.7 ± 1.06
HIV+	Good	17	2.90 ± 0.89	3.19 ± 0.62	4.48 ± 1.13	5.55 ± 0.99
	Poor	10	3.54 ± 1.89	2.91 ± 1.60	5.03 ± 1.66	5.39 ± 1.84
All	Good	47	0.51 ± 0.61	0.75 ± 0.51	2.34 ± 0.68	2.77 ± 0.63
	Poor	23	3.19 ± 0.92	2.89 ± 0.80	4.84 ± 0.90	5.56 ± 0.97
HP	Δ = Good-Poor	43	−3.77^**^	−3.50^**^	−3.57^**^	−4.51^**^
HIV+	Δ = Good-Poor	27	−0.64	0.28	−0.55	0.16
All	Δ = Good-Poor	70	−2.68^*^	−2.14^*^	−2.50^*^	−2.80^*^

Significant differences (t-test,

* p < 0.05,

** *p < 0.01) are marked with an asterisk*.

**Table 3:** Average LPP amplitude for all groups and subgroups based on simulator driving performance.

		***n***	**LPP (Target) Mean** **± SEM (uV)**	
			**Fz**	**F3**
HP	Good	32	3.94 ± 0.70	2.89 ± 0.60
	Poor	16	0.49 ± 1.58	−0.12 ± 1.34
HIV+	Good	19	2.74 ± 0.78	2.76 ± 0.77
	Poor	11	2.56 ± 1.34	2.04 ± 1.61
All	Good	51	3.49 ± 0.52	2.84 ± 0.47
	Poor	27	1.33 ± 1.08	0.75 ± 1.03
HP	Δ = Good-Poor	48	3.45^*^	3.02^*^
HIV+	Δ = Good-Poor	30	0.19	0.72
All	Δ = Good-Poor	78	2.16^*^	2.08^*^

Significant differences (t-test,

* p < 0.05, ^**^p < 0.01) are marked with an asterisk.

### EEG Measures During the Simulator and On-Road Drive

EEG was acquired during the simulated driving scenario as well as the on-road drive in order to identify any possible real-time neurophysiological differences associated with driving performance. However, there were no significant findings.

### EEG and Behavioral Measures in Relation to Cognitive Status

Cognitive status (impaired vs. unimpaired, see section Cognitive and Medical Assessment) was not correlated with any of the behavioral, EEG, and driving performance measures included in this study.

### Classifier for Predicting On-Road Driving Performance

**Figure 10:**
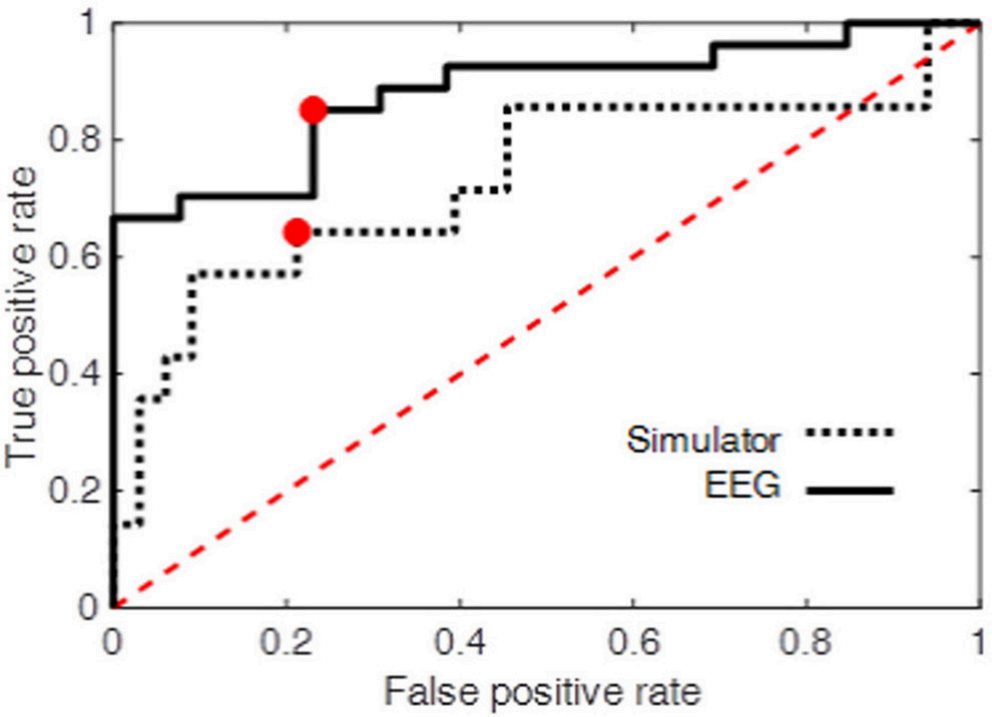
Receiver operating curve (dotted line, simulator; solid line, 3CVT EEG ERP).

At the operating point the true positive rate and false positive rate of the classifier were 0.85 and 0.23, respectively. The area under ROC curve was also used as an overall measure of classification performance. The results (AUC = 0.88) were compared with another LDF using only performance measures obtained from the driving simulator as the predictors (see Driving Simulator Performance) resulting in AUC = 0.73. The true positive and false positive rate at the operating point of this second classifier was 0.64 and 0.21, respectively. Figure 10 shows the ROC curve for both classifiers. The higher performance of the EEG-based classifier, as opposed the classifier based on simulator data, demonstrates the power of EEG measures during an attention task in predicting on-road driving performance.

## Discussion

Evidence from the present study revealed an association between on-road driving performance and EEG ERP data obtained during a short neurocognitive test of sustained attention (3CVT). The 3CVT EEG ERP measures were related to driving performance during a driving simulator task as well as an on-road driving evaluation. Unsafe on-road drivers and Poor drivers in the simulator both exhibited significantly larger P200 amplitude over the left frontal-central region compared to Safe (on-road) and Good (simulator) drivers, respectively. While this finding was observed for Target (frequent) and Non-Target (less frequent) trials, it was largest in response to Non-Target trials during the 3CVT. The P200 component is believed to index automatic, stimulus-driven allocation of attention to stimuli and may reflect biases for preferential processing of particular types of stimuli (Eldar et al., 2010; Gole et al., 2012; McIntosh et al., 2015). In this study, the association between P200 amplitude and driving performance may be linked to deficits in selective attention. Bad drivers exhibit impaired ability to maintain focus, improper allocation of attention, and are more easily distracted. In a separate study in which 3CVT EEG ERP biomarkers were evaluated in patients with a neurodegenerative disease affecting memory (amnestic MCI), no P200 differences were observed compared to healthy controls (Waninger et al., 2018). These amnestic MCI patients did not present with noticeable attentional deficits.

Additionally, Unsafe on-road drivers and Poor drivers in the simulator both exhibited a lower LPP amplitude over the frontal region, particularly for Target trials, compared to Safe (on-road) and Good (simulator) drivers, respectively. The late positive potential (LPP) has been shown to reflect feature evaluation, memory matching, and decision making (Withaar et al., 2000; Reger et al., 2004; Meghdadi et al., under review). Multiple reports suggest reduced amplitude of the LPP is associated with cognitive decline (Schanke and Sundet, 2000; Charlton et al., 2003; Kay et al., 2008; Cysique et al., 2009; Versijpt et al., 2017; Department of Motor Vehicles, 2018a,c; Meghdadi et al., under review) and normal aging (Polich and Corey-Bloom, 2005; Babiloni et al., 2006, 2010; Olichney et al., 2008; López et al., 2014; Ishii et al., 2017). The association of bad driving and reduced amplitude of the LPP reported in the present study is consistent with previous studies that reported a correlation between LPP reduction and severity of cognitive impairment (Polich and Corey-Bloom, 2005; Garn et al., 2014).

The current study included healthy participants (HP) as well as HIV+ participants with well-controlled immune function as a result of antiretroviral therapy. Although current antiretrovirals are increasing the longevity and overall health of HIV+ individuals, HAND is still prevalent and may affect driving performance. The present study included only participants over the age of 55 due to the high likelihood of age-related decline in driving performance. There were no significant differences observed in driving performance between the HIV+ and healthy groups. In fact, the proportion of bad drivers was equivalent for both groups. Bad drivers (Unsafe or Poor) exhibited an increase in P200 amplitude independent of HIV status with highest observed P200 amplitude in HIV+ Unsafe (or Poor) drivers and lowest P200 amplitude in HP Safe (or Good) drivers. Cognitive status as measured by standard neuropsychological testing (see Cognitive and Medical Assessment) did not correlate with P200 amplitude.

Additionally, group differences were observed in the LPP during 3CVT, with the association between bad driving performance and the reduced amplitude of the LPP only significant for the HP group. While bad drivers (Unsafe or Poor) in the HP group show a significant decrease in LPP compared to HP Safe or Good drivers, this reduction was not observed for the HIV+ group. This may be because the LPP has already been significantly reduced as a result of HIV seropositivity (Hillyard et al., 1973; Polich et al., 2000; Olichney et al., 2011; Papaliagkas et al., 2011).

The classifier used both P200 and LPP metrics to predict drivers as either Safe or Unsafe. However, variables selected by the stepwise feature selection and the results from 3CVT ERP data of the present study suggest the P200 is a stable and reliable predictor of driving performance. Preliminary results suggest this P200 effect is consistently observed across other tests of focused and divided attention (Meghdadi et al., under review).

While EEG measures acquired during the 3CVT sustained attention task were highly associated with driving performance, analysis of the EEG measures acquired in the driving simulator and on-road drive did not significantly predict driving performance. The complexity of the driving scenarios and varying driving strategies employed by participants did not allow for precise event locked EEG analyses as was the case for 3CVT. Although participants were instructed to follow the rules of the road, the completion time for each segment of the driving scenario and on-road drive varied widely between participants. The only highly controlled segment of the either task was the SuRT task performed during the simulated driving scenario. SuRT task difficulty was inversely correlated with SuRT driving and secondary task performance. However, neither was correlated with overall simulated or on-road driving performance.

In this study, EEG ERPs observed during attention tasks and their relation to driving performance provide the basis for an inexpensive, fast, and reliable screening exam for elderly drivers using only EEG acquired concurrently during attention tasks. Performance in the driving simulator alone provided only a reasonable prediction of on-road driving performance but was not nearly as accurate as the 3CVT EEG-based classifier.

Driving is an essential aspect of maintaining independence, but driving ability can begin to deteriorate as people age. Through natural aging or disease-related causes, functional impairments can impede elderly drivers from driving safely. ERP measures (P200 and LPP) described in this study are shown to reliably predict driving performance in both healthy and HIV+ individuals across a broad age spectrum (55–87 years old). A diagnosis of a neurodegenerative disease (MCI, PDD, HAND, AD, etc.) alone does not necessarily mean an individual is too impaired to drive safely. In the present study, standard neuropsychological testing was not predictive of driving performance. Currently, there is no sensitive test to determine if an individual is actually impaired except for an on-road drive with a driving examiner. To address this unmet need, a portable EEG system could be used to perform a short and inexpensive neurocognitive test to obtain ERP data for any patient. This ERP data could in turn be fed into a classifier to determine whether or not an individual requires an on-road driving evaluation (classifier responded Unsafe or Safe). While there is a false positive rate of 23%, this approach offers a much better alternative than requiring on-road evaluations for all older or cognitively impaired drivers. Additionally, the model will be improved and refined by increasing the size of the dataset with other populations currently being studied.

Future research is required to fully describe the P200 effect by implementing different types of tasks designed to activate neural circuitry associated with varying aspects of attention and cognition. In the field of driving assessment, further experiments with larger and more diverse populations (including drivers with a variety of neurodegenerative diseases) are needed. A more in-depth analysis of driving performance is also needed to further understand the specific functional deficits associated with increased P200 amplitude.

## Data Availability Statement

A link to download the de-identified data (.edf files) will be made available upon request.

## Ethics Statement

The protocols in the study were approved by both the University of California, San Diego (UCSD) IRB and Sharp IRB (IRBANA) with written informed consent of all subjects. The authors only received de-identified data. HIPPA guidelines were followed throughout the study to protect patient privacy.

## Author Contributions

TM and CB conceived the present project, and CB supervised the project. GR wrote the manuscript with the help of CB, AM, SS, and TM. ES and KM collected the in-lab data, and GR collected the data in the field. AM, MK, MC, and GR analyzed the data. TM, ES, KM, and TR designed and implemented the simulated drive and TM designed the on-road drive. CB, AM, GR, MC, and MK interpreted the results.

### Conflict of Interest Statement

GR, CB, AM, MK, MC, SS are paid salaries or consulting fees by Advanced Brain Monitoring, and CB is a shareholder of Advanced Brain Monitoring, Inc. The remaining authors declare that the research was conducted in the absence of any commercial or financial relationships that could be construed as a potential conflict of interest.
