# Supplementary material for: EEG-Based Neurocognitive Metrics May Predict Simulated and On-Road Driving Performance in Older Drivers
Source: Front Hum Neurosci. 2019 Jan 15;12:532. doi: 10.3389/fnhum.2018.00532 (PMC6341028; doi:10.3389/fnhum.2018.00532)
Supplement: Supplementary file 1 [file Table_1.docx]

Supplementary Material

EEG-Based Neurocognitive Metrics May Predict Simulated and On-Road Driving Performance in Older Drivers

**Greg Rupp^1^, Chris Berka^1^ Amir H. Meghdadi^1^, Marija Karic^1^, Marc Casillas^1^, Stephanie Smith^1^, Theodore Rosenthal^2^, Kevin McShea^3^, Emily Sones^3^, Thomas D. Marcotte^3^**

^1^ Advanced Brain Monitoring Inc. Carlsbad, California, USA, 92008

^2^ Systems Technology, Inc. Hawthorne, CA USA

^3^Department of Psychiatry, University of California San Diego, San Diego, CA, USA 92103

**Correspondence:**Greg Rupp
grupp@b-alert.com

# Supplementary Figures

**Supplementary Figure 1.** On-road scoring sheet (see PDF uploaded with submission)

**Supplementary Table 1**. List of the blocks of the on-road drive as well as the average time each block took to complete

| **Block Names (in Sequential Order)** | **Average Length (seconds)** | **Range (seconds)** |
| --- | --- | --- |
| **Sharp Rehab Parking Lot** | 38 ± 11 | 11 - 60 |
| **Neighborhood Block** | 297 ± 35 | 211 - 414 |
| **Children’s Hospital Block** | 156 ± 42 | 107 - 304 |
| **City Block 1** | 449 ± 116 | 249 - 919 |
| **Target Parking Lot Block** | 206 ± 55 | 58 - 465 |
| **City Block 2** | 270 ± 119 | 149 - 973 |
| **Destination Drive** | 510 ± 99 | 286 - 796 |
| **City Block 3** | 289 ± 68 | 137 - 472 |
| **Freeway Block** | 299 ± 39 | 229 - 465 |
| **Return to Medical Center Block** | 191 ± 55 | 59 – 336 |
| **Medical Center Parking Lot Block** | 47 ± 21 | 11 – 133 |
| **All** | 2705 ± 320 | 1822 – 3643 |

**Supplementary Table 2**. The length and range of time spent in each block during the challenge drive.

| **Challenge Drive Block Names (in Sequential Order)** | **Average Length (Number of Epochs)** | **Range (Number of Epochs)** |
| --- | --- | --- |
| **School Zone** | 38 ± 12.0 | 17 - 81 |
| **Residential Condos** | 161 ± 55.9 | 97 - 338 |
| **SuRT_Easy** | 216 ± 40.1 | 140 - 367 |
| **Suburban_Shops_Amber_Dilemma** | 210 ± 46.3 | 140 - 444 |
| **SuRT_Medium** | 212 ± 36.9 | 137 - 349 |
| **Farmland** | 205 ± 36.6 | 111 - 338 |
| **Big_Rig_Passing** | 134 ± 37.7 | 72 - 257 |
| **City_1** | 181 ± 55.2 | 116 - 487 |
| **City_2** | 256 ± 47.4 | 196 - 494 |
| **Fog_Highway** | 50 ± 20.7 | 27 -114 |
| **SuRT_Hard** | 234 ± 51.0 | 131 - 394 |
| **Hill** | 107 ±7.0 | 84 - 129 |
| **Construction Difficult** | 133 ± 38.1 | 61- 255 |
| **All** | 2143 ± 324.3 | 1674 - 3245 |

**Supplementary Table 3**. P200 and LPP ERP measurements (Safe minus Unsafe) obtained during 3CVT for all groups and all channels for both Target and NonTarget trials

| **ERP Component** | **Group** | **Target or NonTarget** | **POz** | **Fz** | **Cz** | **C3** | **C4** | **F3** | **F4** | **P3** | **P4** |
| --- | --- | --- | --- | --- | --- | --- | --- | --- | --- | --- | --- |
| LPP mean | HC | Target | 2.12 (p=0.19) | 4.28 (p=0.08) | 3.25 (p=0.14) | 2.96 (p=0.09) | 2.71 (p=0.12) | 4.64 (p=0.03) | 4.09 (p=0.07) | 2.31 (p=0.11) | 2.07 (p=0.19) |
| LPP mean | HIV | Target | 0.56 (p=0.63) | 0.39 (p=0.81) | 0.14 (p=0.93) | -0.8 (p=0.55) | 0.07 (p=0.96) | -1.31 (p=0.54) | 0.29 (p=0.87) | -0.38 (p=0.74) | 0.15 (p=0.9) |
| LPP mean | ALL | Target | 1.44 (p=0.15) | 2.3 (p=0.13) | 1.7 (p=0.22) | 1.08 (p=0.33) | 1.63 (p=0.16) | 1.61 (p=0.28) | 2.38 (p=0.1) | 1.04 (p=0.26) | 1.33 (p=0.18) |
| LPP mean | HC | NonTarget | 2.28 (p=0.26) | 3.17 (p=0.16) | 2.59 (p=0.3) | 1.45 (p=0.4) | 2.93 (p=0.16) | 1.42 (p=0.44) | 4.02 (p=0.07) | 2.11 (p=0.24) | 2.63 (p=0.2) |
| LPP mean | HIV | NonTarget | -1.39 (p=0.35) | 1.36 (p=0.54) | -1.06 (p=0.62) | -0.57 (p=0.78) | -0.07 (p=0.97) | 0.54 (p=0.79) | 0.3 (p=0.91) | -1.41 (p=0.38) | -1.66 (p=0.26) |
| LPP mean | ALL | NonTarget | 0.83 (p=0.52) | 2.12 (p=0.17) | 0.84 (p=0.61) | 0.39 (p=0.76) | 1.67 (p=0.26) | 0.81 (p=0.54) | 2.41 (p=0.15) | 0.61 (p=0.61) | 0.91 (p=0.49) |
| P200 avg | HC | Target | 1.08 (p=0.44) | -2.44 (p=0.11) | -2.92 (p=0.06) | -1.24 (p=0.26) | -2.51 (p=0.04) | -0.88 (p=0.5) | -2.55 (p=0.06) | 0.44 (p=0.69) | -0.39 (p=0.76) |
| P200 avg | HIV | Target | -1.62 (p=0.31) | -0.22 (p=0.93) | -2.43 (p=0.31) | -2.35 (p=0.24) | -0.9 (p=0.66) | -2.35 (p=0.4) | 0.82 (p=0.76) | -1.96 (p=0.18) | -1.24 (p=0.4) |
| P200 avg | ALL | Target | -0.41 (p=0.69) | -1.53 (p=0.25) | -2.92 (p=0.03) | -2.13 (p=0.05) | -1.77 (p=0.1) | -1.9 (p=0.17) | -0.95 (p=0.48) | -0.91 (p=0.3) | -0.88 (p=0.35) |
| P200 avg | HC | NonTarget | 0.45 (p=0.78) | -3.18 (p=0.1) | -3.83 (p=0.04) | -2.62 (p=0.08) | -2.08 (p=0.15) | -2.96 (p=0.14) | -1.89 (p=0.31) | -0.36 (p=0.81) | -0.5 (p=0.73) |
| P200 avg | HIV | NonTarget | -2.38 (p=0.27) | 0.71 (p=0.8) | -2.02 (p=0.48) | -1.6 (p=0.5) | -1.35 (p=0.62) | -0.28 (p=0.92) | 0.37 (p=0.91) | -2.19 (p=0.24) | -1.84 (p=0.37) |
| P200avg | ALL | NonTarget | -0.93 (p=0.46) | -1.78 (p=0.26) | -3.35 (p=0.03) | -2.59 (p=0.05) | -1.88 (p=0.16) | -2.2 (p=0.19) | -1 (p=0.54) | -1.41 (p=0.22) | -1.15 (p=0.31) |

**Supplementary Table 4**. P200 and LPP ERP measurements (Good minus Poor) obtained during 3CVT for all groups and all channels for both Target and NonTarget trials

| **ERP Component** | **Group** | **Target or NonTarget** | **POz** | **Fz** | **Cz** | **C3** | **C4** | **F3** | **F4** | **P3** | **P4** |
| --- | --- | --- | --- | --- | --- | --- | --- | --- | --- | --- | --- |
| LPP mean | HC | Target | 2.16 (p=0.04) | 3.45 (p=0.03) | 2.86 (p=0.04) | 2.11 (p=0.06) | 2.37 (p=0.03) | 3.02 (p=0.02) | 3.14 (p=0.03) | 1.89 (p=0.04) | 1.8 (p=0.07) |
| LPP mean | HIV | Target | -0.63 (p=0.44) | 0.38 (p=0.79) | -0.83 (p=0.56) | -0.74 (p=0.5) | -0.14 (p=0.91) | 0.36 (p=0.82) | 0.54 (p=0.72) | -0.78 (p=0.34) | -0.68 (p=0.41) |
| LPP mean | ALL | Target | 1.09 (p=0.13) | 2.2 (p=0.04) | 1.4 (p=0.17) | 0.96 (p=0.23) | 1.42 (p=0.08) | 1.88 (p=0.06) | 2.13 (p=0.04) | 0.85 (p=0.19) | 0.87 (p=0.21) |
| LPP mean | HC | NonTarget | 0.65 (p=0.64) | 1.55 (p=0.33) | 1.43 (p=0.39) | 0.32 (p=0.79) | 2.35 (p=0.08) | -0.16 (p=0.9) | 2.97 (p=0.05) | 0.62 (p=0.61) | 1.6 (p=0.24) |
| LPP mean | HIV | NonTarget | -1.66 (p=0.11) | -0.64 (p=0.75) | -2.62 (p=0.18) | -1.2 (p=0.44) | -1.61 (p=0.36) | -0.92 (p=0.61) | -0.84 (p=0.69) | -1.31 (p=0.2) | -1.56 (p=0.13) |
| LPP mean | ALL | NonTarget | -0.2 (p=0.83) | 0.64 (p=0.6) | -0.19 (p=0.88) | -0.3 (p=0.75) | 0.76 (p=0.48) | -0.52 (p=0.62) | 1.43 (p=0.24) | -0.09 (p=0.91) | 0.39 (p=0.68) |
| P200 avg | HC | Target | 0.07 (p=0.94) | -0.3 (p=0.77) | -1.44 (p=0.19) | -0.76 (p=0.41) | -0.66 (p=0.41) | -0.14 (p=0.89) | 0.05 (p=0.95) | -0.13 (p=0.87) | 0.29 (p=0.72) |
| P200 avg | HIV | Target | -1.74 (p=0.14) | -0.52 (p=0.76) | -1.37 (p=0.42) | -0.96 (p=0.5) | -0.54 (p=0.7) | -0.57 (p=0.76) | 0.39 (p=0.83) | -1.04 (p=0.36) | -1.9 (p=0.07) |
| P200 avg | ALL | Target | -0.71 (p=0.34) | -0.45 (p=0.63) | -1.5 (p=0.11) | -0.94 (p=0.24) | -0.64 (p=0.38) | -0.41 (p=0.67) | 0.18 (p=0.84) | -0.53 (p=0.41) | -0.61 (p=0.34) |
| P200 avg | HC | NonTarget | -0.76 (p=0.51) | -3.57 (p=0.01) | -3.77 (p=0) | -3.52 (p=0) | -1.26 (p=0.25) | -4.51 (p=0) | -1.28 (p=0.37) | -1.45 (p=0.17) | 0.01 (p=1) |
| P200 avg | HIV | NonTarget | -1.41 (p=0.37) | -0.55 (p=0.78) | -0.64 (p=0.73) | 0.28 (p=0.85) | -1.06 (p=0.56) | 0.16 (p=0.93) | -0.37 (p=0.86) | -0.1 (p=0.94) | -1.79 (p=0.23) |
| P200 avg | ALL | NonTarget | -1.08 (p=0.24) | -2.5 (p=0.04) | -2.68 (p=0.02) | -2.15 (p=0.02) | -1.29 (p=0.18) | -2.8 (p=0.02) | -1 (p=0.4) | -0.96 (p=0.25) | -0.76 (p=0.35) |
