# Supplementary material for: EEG-Based Neurocognitive Metrics May Predict Simulated and On-Road Driving Performance in Older Drivers
Source: Front Hum Neurosci. 2019 Jan 15;12:532. doi: 10.3389/fnhum.2018.00532 (PMC6341028; doi:10.3389/fnhum.2018.00532)
Supplement: Supplementary file 2 [file Data_Sheet_1_v1.PDF]

On-Road Evaluation Scoring Form

(Revised 08/30/2016)

Study Number: ____ ____ ____ ____ ____ **Start Time** _____ : _____

Date: ___ ___ / ___ ___ / ___ ___ **End Time** _____ : _____

Verbal Directions	Route	Item #	Scored (detail)	P	F
"Pull away from the curb."	Sharp Parking Lot	1	Turn Signal	0	1
		2	Traffic Check	0	1
		3	Steering Control	0	1
		4	Smooth Acceleration	0	1
"Make a Right turn at the fire hydrant and exit the driveway turning to the Right"	Parking Lot w/Right on Meadowlark	5	Turn Signal	0	1
		6	Traffic/Pedestrian Check	0	1
		7	Stop at Sidewalk	0	1
		8	Steering Control	0	1
		9	Smooth Acceleration	0	1
		10	Speed Control	0	1
"Go straight at the first Stop sign."	Meadowlark & Vista Hill Stop Sign	11	Full Stop	0	1
		12	Gap and Limit Line	0	1
		13	Traffic Check	0	1
		14	Lane Position	0	1
		15	Smooth Acceleration	0	1
		16	Speed Control	0	1
"Continue straight ahead."	Meadowlark & Starling Stop Sign	17	Full Stop	0	1
		18	Gap and Limit Line	0	1
		19	Traffic Check	0	1
		20	Lane Position	0	1
		21	Smooth Acceleration	0	1
		22	Speed Control	0	1
"Turn left on Blue Jay."	Search and Make Unprotected Left on Blue Jay	23	Identifies Turn	0	1
		24	Signal Timely	0	1
		25	Traffic Check	0	1
		26	Rearview Mirror Check	0	1
		27	Lane Position	0	1
		28	Speed Control	0	1
"Turn Right on Meadowlark."	Unprotected Right on Meadowlark	29	Identifies Street	0	1
		30	Signal Timely	0	1
		31	Rearview Mirror Check	0	1
		32	Lane Position	0	1
		33	Speed Control	0	1
"Continue on Meadowlark."		34	Lane Position	0	1
		35	Full Scanning	0	1
"At the next two Stop Sign, continue straight ahead."	1) Starling / Meadowlard	36	Full Stop	0	1
	Stop	37	Gap and Limit Line	0	1
	2) Vista Hill / Meadowlark Stop	38	Traffic Check	0	1
	Stop	39	Lane Position	0	1
	**Score for both Stops**	40	Smooth Accelerate	0	1
		41	Speed Control	0	1

"Follow this street and pass Children's Hospital."		42	Slows to Safe Speed Within 5 MPH of Speed Limit Sign	0	1
		43	Lane Position	0	1
		44	Traffic Awareness	0	1
		45	Pedestrian Awareness	0	1
		46	Scans at Crosswalk and Takes Appropriate Action	0	1
"Continue straight ahead at the Stop Sign."	Frost/Berger Stop	47	Full Stop	0	1
		48	Gap and limit line	0	1
		49	Traffic Check	0	1
		50	Lane Position	0	1
		51	Smooth Accelerate	0	1
		52	Speed Control	0	1
"At the traffic light, there are two lanes that turn Right. Stay in the lane on the Left side to complete your Right turn."	Right turn at intersection<br>Light At Mesa<br>College / Berger	53	Selects Correct Lane	0	1
		54	Signal Timely	0	1
		55	Through Traffic Check	0	1
		56	Turns Into Correct Lane	0	1
		57	Speed Control	0	1
"Continue straight ahead and turn Right at the third traffic light. It comes up quickly."	Kearny Mesa with Right Turn at Aero	58	Counts Intersection Lights	0	1
		59	Identifies Aero Drive	0	1
		60	Signal Timely	0	1
		61	Right curb alignment	0	1
		62	Red Light - Full Stop	0	1
		63	Red Light - Limit Line	0	1
		64	Thorough Scan	0	1
		65	Green Light - Slows to Safe Speed	0	1
		66	Thorough Scan	0	1
		67	Safe Turn	0	1
"When it's safe make a legal U turn and go back."	U turn choices on Aero Drive:<br>1) Aero Court<br>2) Alton/Curtis<br>3) Brookstone	68	Proper Lane Change	0	1
		69	Thorough Search for Safe U Turn Street	0	1
		70	Speed Adjustment	0	1
		71	Proper Lane Change	0	1
		72	Lane Position	0	1
		73	Signal Timely	0	1
		74	Scan Prior U Turn	0	1
"Your next turn is Right on Convoy."	Aero with Right on Convoy	75	Signal Timely	0	1
		76	Right curb alignment	0	1
		77	Red Light - Full Stop	0	1
		78	Red Light - Limit Line	0	1
		79	Thorough Scan	0	1
		80	Green Light - Slows to Safe Speed	0	1
		81	Thorough Scan	0	1
		82	Safe Turn	0	1
"Turn Right on Kearny Mesa Road."		83	Identifies Kearny Mesa	0	1
		84	Signal Timely	0	1
		85	Right curb alignment	0	1
		86	Red Light - Full Stop	0	1
		87	Red Light - Limit Line	0	1
		88	Thorough Scan	0	1
		89	Safe Turn	0	1
"Go to the Target and park in the lot as though you are	**(Can Use Either Drive Entry)**	90	Identifies Target	0	1
		91	Signal Timely	0	1
		92	Pulls into turn lane	0	1

going shopping."	Entry)	93	Thorough Scan	0	1
		94	Safe Turn	0	1
"Back out of this spot and pass in front of Costco and turn Right onto Convoy"		95	Backs out Safely	0	1
		96	Traffic Awareness	0	1
		97	Pedestrian Awareness	0	1
		98	Speed Control	0	1
		99	Stops at Sidewalk	0	1
		100	Thorough Scan	0	1
		101	Safe Pullout	0	1
"A little past Balboa you'll be making a Left Turn."		102	Timely Lane Change Choice	0	1
		103	Signal Timely	0	1
		104	Traffic Check	0	1
		105	Spacing	0	1
		106	Speed Adjustment	0	1
		107	Smooth Transition	0	1
		108	Steering Control	0	1
"Make a Left turn on Ronson Road."		109	Identifies Ronson Road	0	1
		110	Signal	0	1
		111	Traffic Check	0	1
"See the Wendy's? We are going to drive several streets away from here. Remember the route. You will make your way back to Wendy's without instruction using the same streets."					
"Turn Right at Ruffner."	Right on Ruffner	112	Full Stop	0	1
		113	Traffic Check	0	1
		114	Signal	0	1
		115	Curb Alignment	0	1
"Turn Left on Clairemont Mesa."	Left on Clairemont Mesa	116	Signal	0	1
		117	Adhere to Interesection Light	0	1
		118	Lane Maintenance	0	1
"Turn Left on Shawline."	Left on Shawline	119	Signal	0	1
		120	Adhere to Intersection Light	0	1
		121	Lane Maintenance	0	1
"Find the Food 4 Less and Make a Left Turn Into the Parking Lot."	Find Food 4 Less	122	Safe Lane Management	0	1
		123	Monitors Mirrors When Slowing	0	1
		124	Identifies Food for Less	0	1
		125	Enters Food for Less Safely	0	1
		126	Scans Parking Lot	0	1
		127	Speed Control	0	1
"Now, return to Wendy's backtraking the same route."	Right on to Shawline	128	Full Scan	0	1
		129	Head Turn	0	1
		130	Exits Driveway Safely	0	1
		131	Turns Correct Direction	0	1
No Instructions	Right on to Clairemont Mesa	132	Full Stop	0	1
		133	Signal	0	1
		134	Multiple Traffic Checks	0	1
		135	Chooses Correct Street	0	1
No Instructions	Right on to Ruffner	136	Full Stop	0	1
		137	Signal	0	1
		138	Multiple Traffic Checks	0	1

		139	Chooses Correct Street	0	1

No Instructions	Left on to Ronson	140	Full stop	0	1
		141	Signal	0	1
		142	Multiple Traffic Checks	0	1
		143	Chooses Correct Street	0	1
		144	Finds Final Destination	0	1
"After you turn into Wendy's park the car."		145	Signal	0	1
		146	Traffic Check	0	1
		147	Stall Choice	0	1
		148	Stall Position	0	1
		149	Full Scan	0	1
		150	Backing out Safely	0	1
"Exit the driveway you entered and turn to the Right. At Convoy, make a Left turn."	Ronson Turn	151	Signal	0	1
		152	Traffic Check	0	1
		153	Stops at Sidewalk	0	1
		154	Yield to Traffic	0	1
	Convoy Turn	155	Signal	0	1
		156	Traffic Check	0	1
		157	Yeilds to Oncoming Traffic	0	1
"Find the 52 freeway and go East."	Convoy/52 East	158	Identifies Onramp	0	1
		159	Chooses Correct Lane	0	1
		160	Safe Lane CX PRN	0	1
"Exit on the 163 Freeway South."		161	Identifies 163	0	1
		162	Position Correct Lane	0	1
		163	Traffic Check	0	1
		164	Signal	0	1
		165	Speed Control	0	1
"Take the Balboa exit East."		166	Exits Freeway Safely	0	1
"Now, Merge Back onto the Freeway."		167	Chooses Correct Lane	0	1
		168	Enters Freeway Thorough Scanning	0	1
		169	Good Speed	0	1
		170	Safe Merge	0	1
"Exit on Genesee and turn Left."	Genesse	171	Traffic Check	0	1
		172	Correct Lane Change to Exit on Genesse	0	1
		173	Signal	0	1
		174	Speed Control	0	1
		175	Signal	0	1
"In two traffic lights, turn Left on Healthcenter Road."	Healthcenter	176	Signal	0	1
		177	Traffic Check	0	1
		178	Correct Lane	0	1
		179	Identifies HC Drive	0	1
"Turn Right into the first Driveway to Sharp Rehabilitation."		180	Identifies Driveway	0	1
		181	Signal	0	1
		182	Speed Control	0	1
		183	Pedestrian Check	0	1
		184	Traffic Check	0	1
"Pull up to the curb in front of the Rehabilitation Center."		185	Maneuver Through Parking Lot With No Extra Clues	0	1
		186	Parks Safely on Curb	0	1

**CRITICAL ERRORS:**		**NUMBER**
187. Physical Intervention by Examiner	____________________	______
188. Verbal Intervention by Examiner	____________________	______
189. Strikes Object	____________________	______
190. Disobeys Traffic Sign or Signals	____________________	______
191. Disobeys Safety Personnel	____________________	______
192. Dangerous Maneuver	____________________	______
193. Speed Verbal Cues	____________________	______
194. Lane Violation	____________________	______

EXAMINER SCORE

195.	1	2	3	4	5
	Excellent	Good	Marginal	Recommend Intervention	Should not be Driving

CONSENSUS SCORE

197.	1	2	3	4	5
	Excellent	Good	Marginal	Recommend Intervention	Should not be Driving

198. **NOTES:** ____
